# How man-made interference might cause gas bubble emboli in deep diving whales

**DOI:** 10.3389/fphys.2014.00013

**Published:** 2014-01-28

**Authors:** Andreas Fahlman, Peter L. Tyack, Patrick J. O. Miller, Petter H. Kvadsheim

**Affiliations:** ^1^Department of Life Sciences, Texas A&M University Corpus ChristiCorpus Christi, TX, USA; ^2^Sea Mammal Research Unit, University of St. AndrewsSt. Andrews, UK; ^3^Maritime Systems Division, Norwegian Defence Research Establishment (FFI)Horten, Norway

**Keywords:** modeling, cetacean, diving physiology

## Abstract

Recent cetacean mass strandings in close temporal and spatial association with sonar activity has raised the concern that anthropogenic sound may harm breath-hold diving marine mammals. Necropsy results of the stranded whales have shown evidence of bubbles in the tissues, similar to those in human divers suffering from decompression sickness (DCS). It has been proposed that changes in behavior or physiological responses during diving could increase tissue and blood N_2_ levels, thereby increasing DCS risk. Dive data recorded from sperm, killer, long-finned pilot, Blainville's beaked and Cuvier's beaked whales before and during exposure to low- (1–2 kHz) and mid- (2–7 kHz) frequency active sonar were used to estimate the changes in blood and tissue N_2_ tension (P_N_2__). Our objectives were to determine if differences in (1) dive behavior or (2) physiological responses to sonar are plausible risk factors for bubble formation. The theoretical estimates indicate that all species may experience high N_2_ levels. However, unexpectedly, deep diving generally result in higher end-dive P_N_2__ as compared with shallow diving. In this focused review we focus on three possible explanations: (1) We revisit an old hypothesis that CO_2_, because of its much higher diffusivity, forms bubble precursors that continue to grow in N_2_ supersaturated tissues. Such a mechanism would be less dependent on the alveolar collapse depth but affected by elevated levels of CO_2_ following a burst of activity during sonar exposure. (2) During deep dives, a greater duration of time might be spent at depths where gas exchange continues as compared with shallow dives. The resulting elevated levels of N_2_ in deep diving whales might also make them more susceptible to anthropogenic disturbances. (3) Extended duration of dives even at depths beyond where the alveoli collapse could result in slow continuous accumulation of N_2_ in the adipose tissues that eventually becomes a liability.

## Introduction

A reduction in pressure results in decreased gas solubility. Once the dissolved tissue gas tension (P_tiss_) exceeds the ambient pressure (P_amb_), the tissue is supersaturated, and bubbles may form. The bubbles are believed to be the instigator for **decompression sickness (DCS)** symptoms seen in human divers, or gas bubble emboli found in marine mammals (Moore et al., 2009; Bernaldo De Quirós et al., 2012; Dennison et al., 2012). Scholander (1940) hypothesized that the unusual respiratory system in marine mammals, with a stiff trachea and rather compliant chest, allows the alveoli to collapse at shallow depths, thereby limiting the uptake of inert gas, reducing the level of N_2_ that would be taken up during a dive and therefore reducing the likelihood of gas bubble emboli in breath-hold diving mammals. However, necropsy results in stranded marine mammals have indicated lesions that are similar to those found in human divers with DCS symptoms (Jepson et al., 2003; Fernández et al., 2005). Also more recent work has suggested that marine mammals may experience inert gas bubbles more commonly than formerly thought (Bernaldo De Quirós et al., 2012; Dennison et al., 2012; Hooker et al., 2012). Consequently, cetaceans do not appear immune against gas bubble emboli. However, our understanding of their natural dive behavior and physiology is limited and the following review highlights recent work that aims to estimate how changes in dive behavior could affect the risk of gas bubble emboli.

KEY CONCEPT 1. Decompression sickness (DCS) Dissolved gas coming out of solution and forming bubbles during a reduction in pressure. The bubbles may form in all tissues, but in certain critical tissues such as the central nervous system (CNS), the damage can cause serious lesions. In the blood, the bubbles may embolize blood vessels leading to severe ischemic damage.

The alveolar/lung collapse hypothesis formulated by Scholander (1940) implies that marine mammals that spend a significant time just above the depth of alveolar collapse depth should have the highest levels of absorbed N_2_ and therefore the highest risk of gas bubble emboli. Recent work by Bernaldo De Quirós et al. (2012) showed that among stranded whales, deep diving species of whales, such as sperm and beaked whales, had a higher abundance of gas bubbles compared to shallow diving species. The bubbles were mainly composed of N_2_, confirming that the bubbles were related to DCS and not caused by putrefaction. Gas exchange modeling using dive records from three species of deep diving beaked whales (Table 3 in Hooker et al., 2009) found that these deep diving cetaceans could have very high mixed venous P_*N*_2__ (P_venN_2__) at the end of the dive (P_venN_2__ > 2 ATA) following deep dives and high tissue P_N_2__ (P_tissN_2__) at depth (P_tissN_2__ > 3 ATA). Anthropogenic acoustic disturbances such as naval **sonar**, have been shown to alter the dive pattern of cetacean deep divers (Tyack et al., 2011; Sivle et al., 2012; Deruiter et al., 2013). In a recent comparative study, using the same gas exchange model as Hooker et al. (2009), Kvadsheim et al. (2012) compared estimated blood and tissue P_N_2__ levels in shallow (killer whales), intermediate (pilot whales) and deep diving cetaceans (beaked whales and sperm whales). The results indicated that even though deep divers spend significant part of the dives below the expected depth of alveolar collapse, where there is no gas exchange, they have higher end-dive blood and tissue N_2_ levels, and therefore a higher risk of developing gas bubble emboli, as compared with shallow diving species. This focused review tries to explain why deep divers have a higher risk of developing decompression related symptoms. We propose three possible explanations: (1) Elevated blood and tissue CO_2_ levels from metabolism may instigate bubble growth. (2) During transit to depth and during shallower dives, deep divers spend an accumulated greater duration at depths where gas exchange continues compared to shallow divers. (3) The longer duration of deep dives allows redistribution of N_2_ from fast (muscle, heart) to slow tissues (adipose).

KEY CONCEPT 2. Sonar The use of sound to detect obstacles (other vessels), to help navigate or communication underwater. Often used by submarines or to detect submarines.

## Materials and methods

We used a previously published and calibrated gas exchange model (Fahlman et al., 2009; Hooker et al., 2009) to predict blood and tissue N_2_ tension (P_*N*_2__) from the dive behavior of five species of large whales (killer whale, pilot whale, sperm whale, Cuvier's beaked whale, and Blainville's beaked whale) before and during exposure to naval sonar (Kvadsheim et al., 2012). Metabolic gases (O_2_, CO_2_) and the inert gas N_2_ are exchanged according to partial pressure gradients from the lungs into the circulatory system and to 4 tissue compartments (central circulation, muscle, brain, and fat). Bubble formation and growth in tissues and blood can occur when the gas tissue tension exceeds the ambient partial pressure. As the P_venN_2__ is an estimate of the mean P_tissN_2__ of the animal, we assumed that the risk of gas bubble emboli following a dive increased with the mixed venous **supersaturation** (P_venN_2__—P_ambN_2__).

KEY CONCEPT 3. Supersaturation When the tissue or blood gas tension exceeds the ambient (hydrostatic) pressure. The probability of bubble formation increases with increasing supersaturation. Some level of supersaturation may be tolerated, but current research suggests that any hyperbaric exposure has a finite probability of DCS.

## Results

**Figure 1:**
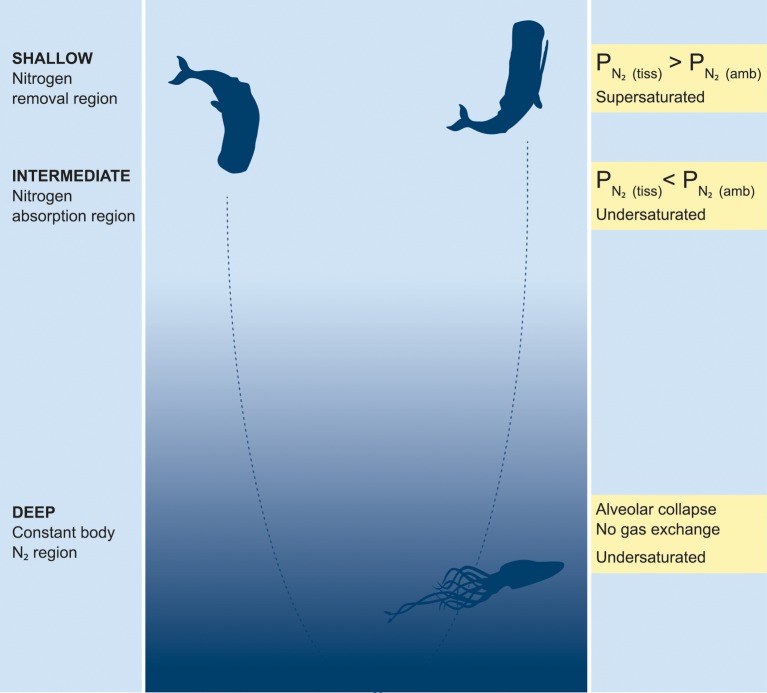
**Conceptual model of risk of gas embolism in deep diving mammals**. The descent and ascent of deep dives goes through three different depth ranges representing different risk of gas bubble embolism (Kvadsheim et al., 2012). The *Shallow N_2_ removal* region where N_2_ is excreted because the partial pressure of N_2_ in the tissue is higher than the ambient hydrostatic pressure [P_N_2(tiss)__ > P_N_2(amb)__], the *Intermediate N_2_ absorption* region where N_2_ is absorbed because the ambient partial pressure of N_2_ is higher than in the tissue [P_N_2(tiss)__ < P_N_2(amb)__], and the *Deep constant body N_2_* region where N_2_ is neither absorbed nor excreted in the lungs (no gas exchange) because of alveolar collapse. The gas exchange model used in this paper suggests that the *Deep constant body N_2_* region starts when the alveoli are completely collapsed at 180–220 m, depending on species, and extend downward as deep as the animal might dive. The depth range of the *Shallow N_2_ removal* region (decompression zone) will constantly vary depending on the P_N_2__ level of the tissue, and thus the dive history of the animal. It might be restricted to just the surface or extend down to depths of 20–30 m if the animal has a high P_N_2__ level built up during previous dives. The risk of gas bubble embolism depends on the **saturation** level, which is determined by the P_N_2__in the tissue and the hydrostatic pressure. N_2_ levels in the tissue will increase if more time is spent in the *Intermediate N_2_ absorption* region and less time in the *Shallow N_2_ removal* region. Time spent in the *Deep constant body N_2_* region, does not add to the total body nitrogen, but the extended duration of the deep dives allows for transfer of N_2_ into poorly perfused slow tissues such as blubber, where it might accumulate to dangerous levels. Gas embolism will only occur in supersaturated tissues and because this requires a low hydrostatic pressure, it only occurs in the *Shallow N_2_ removal* region, including the surface. Thus, the *Intermediate N_2_ absorption* region is where N_2_ is absorbed, but the *Shallow N_2_ removal* region is what represents the immediate risk of gas embolism. See text for further details Illustration IAN, FFI.

**Figure 2:**
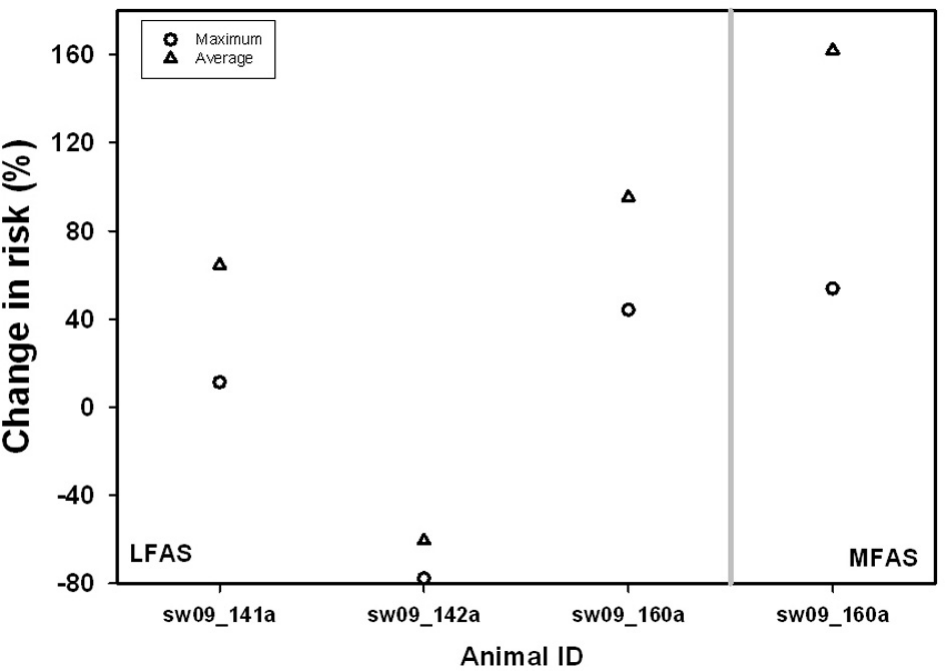
**Average (Δ) and maximum (o) change in risk {*risk* = [P_venN_2__ − P_N__2_(amb)__]} during 1–2 kHz LFAS (left) or 6–7 kHz MFAS (right) sonar exposure compared to control in behaviorally responding sperm whales**. Data are modified from Kvadsheim et al. (2012).

In general, there was a positive relationship between the end-dive P_tissN_2__ and P_venN_2__ with depth of dive, suggesting higher supersaturation in the deep divers (sperm whales and beaked whales) than in the shallower divers (killer whales; see Figure 2 in Kvadsheim et al., 2012). The model output was used to estimate 3 depth regions for diving vertebrates. While the depth of each region will vary depending on the specific dive profile and parameters used, the regions provide a conceptual model to help explain variation in risk between species. In the current study a depth of 30 m was used for shallow dives where P_tissN_2__ > P_ambN_2__, a depth of 200 m for alveolar collapse (deep dives), and an intermediate dive range (>30 and <200 m) (Figure 1). The largest increase occurred for dives from shallow (<30 m) to intermediate (>30 and <200 m) dive depths with only a minimal additional increase for dives >200 m (Figure 1). All species showed some changes in dive behavior during sonar exposure (Tyack et al., 2011; Sivle et al., 2012; Deruiter et al., 2013), but only in sperm whales did this behavioral change result in increased risk of DCS in 3 out of 4 animals (Figure 2).

KEY CONCEPT 4. Saturation The amount of gas dissolved in tissue or blood is a function of the pressure and duration of the changed pressure. The tension of dissolved gas continues to increase until equilibrium with the environment occurs, at which time the tissue or blood is said to be saturated.

## Discussion

Our model estimates suggest that shallow (killer whales), intermediate (pilot whales), and deep diving whales (sperm whale, Cuvier's beaked whale, and Blainville's beaked whale) all live with high blood and tissue *P*_*N*_2__ levels (>2 ATA), with the deep diving whales having the most extreme values (Kvadsheim et al., 2012). When a diver is exposed to elevated pressure, the body takes up additional gas according to the partial pressure gradients. As the diver returns toward the surface, the gas solubility decreases, and when the blood or tissue gas tension exceeds the ambient pressure the blood or tissue becomes supersaturated, and bubbles may form and grow. Nitrogen is inert and must be returned to the lungs to be exhaled. As O_2_ is continuously consumed in aerobic metabolism, it is not considered to be involved in DCS. Carbon dioxide, on the other hand, is produced by aerobic metabolism and may accumulate to high levels in metabolically active tissues. Local accumulation, and high diffusivity of CO_2_ may form bubble precursors that continues to grow from N_2_ diffusion (Harris et al., 1945; Behnke, 1951; Bernaldo De Quirós et al., 2012, 2013). Even though the carbonic anhydrase enzyme system might dissolve such bubble precursors quickly, they might still trigger bubble formation in N_2_-supersaturated tissue. These characteristics suggest that CO_2_ may play an unrecognized role in the formation of bubbles and the initial growth of gas emboli. Thus, elevated levels of CO_2_ following a burst of activity may initiate the growth of the bubble with diffusion of N_2_ into the bubble as the supersaturation increases close to the surface. Generally, deep divers also make the longest duration dives and are therefore expected to have the highest tension of CO_2_ when they surface.

Necropsy results from stranded cetaceans have shown a greater bubble density in deep diving species (Bernaldo De Quirós et al., 2012), which agrees with the theoretical results of Kvadsheim et al. (2012) that deep divers should be at higher risk of developing bubbles. The gas composition of the bubbles was mainly N_2_, but there were also high levels of CO_2_. The authors suggested that CO_2_ may initiate bubble formation and growth, while elevated levels of N_2_ may be important for continued bubble growth (Bernaldo De Quirós et al., 2012). This hypothesis has some interesting implications as CO_2_ production continues throughout the dive and is independent of the alveolar collapse depth. If so, the formation and initial growth of bubbles may be related to the *P*_CO_2__. According to this hypothesis, alveolar collapse may exacerbate the risk of bubble formation as the lung would be unavailable as a sink for the CO_2_ and the *P*_CO_2__ would increase in the blood. In addition, if CO_2_ is important for bubble genesis, the activity and overall metabolic rate of the whale during a dive would also affect the risk (Fahlman et al., 2013). A plausible scenario is a cetacean that exerts more energy to escape a sound source. The increased metabolic rate, CO_2_ production, and alteration in cardiac output could thereby increase the risk of gas bubble emboli.

Variation in body size and physiology between and within whale species makes it difficult to predict which dive behaviors constitute the highest risk of gas bubble embolism. However, based on our current knowledge of physiology we have attempted to divide the water column into 3 different zones and the relative time spent in each of these may alter risk (Figure 1). Shallow dives to depths where the tissues become supersaturated imply that N_2_ is removed from the tissues (Fahlman et al., 2007). The actual depth where the transfer of N_2_ switches from being taken up by tissues to being removed from tissues, i.e., the supersaturation depth, depends on P_tissN_2__ and will change between compartments, dives, and species. Based on the results of Hooker et al. (2009) it seems as if mixed venous P_*N*_2__ does not exceed 2.8 ATA in diving beaked whales, and this corresponds to a depth of approximately 30 m. The likelihood for bubbles to form is positively correlated with the supersaturation level (Gerth and Vann, 1997). Thus, shallow dives may serve an important purpose to safely remove tissue N_2_. As long as P_venN_2__ > P_amb_, N_2_ is removed while the increased P_amb_ by diving shallow reduces the supersaturation and thereby the likelihood for bubble formation. Consequently, dives to depths ranging from 1 to 30 m may have an important function in reducing supersaturation and therefore deserve special attention.

During intermediate dives (30–200 m) pulmonary gas exchange still occurs (Hooker et al., 2009), but the lung P_N_2__ exceeds P_tissN_2__ and P_venN_2__, and N_2_ is taken up. However, the diffusion across the alveolar membrane is complex as the depth related pulmonary shunting begins to impede gas exchange (Kooyman and Sinnett, 1982; Bostrom et al., 2008; McDonald and Ponganis, 2012). While variation in the depth where the alveoli collapse and gas exchange cease may vary with structural properties of the respiratory system (compliance of lungs and trachea) and behavior (diving lung volume adjustment), the rate of diffusion should be significantly reduced for all species at a depth >200 m (Bostrom et al., 2008; Hooker et al., 2009). Variation in dive behavior and physiological responses, e.g., cardiac output (Fahlman et al., 2006; Kvadsheim et al., 2012; Noren et al., 2012) may cause large variation in end-dive tissue and blood P_*N*_2__ in this depth zone (Figure 1). Consequently, the total body N_2_ load will be determined by the ratio of time spent at shallow and intermediate depths, whereas time spent at depths >200 m should not add to the total body N_2_ load.

It has also been suggested that compression and collapse of the alveolar space, and a reduction in cardiac output reduces gas exchange and the inert gas burden (Scholander, 1940). Thus, once a whale is below the depth of alveolar collapse, gas exchange ceases and no more N_2_ is taken up. Theoretical gas exchange modeling has suggested that this should protect deep diving species, but they should only spend a short period in the region where gas is exchanged during ascent and descent (Zimmer and Tyack, 2007). Other theoretical studies, using different assumptions on how pressure affects gas exchange, suggest that diving deeper may increase the risk (Hooker et al., 2009) and that certain anatomical differences or behavioral or physiological responses may further exacerbate the risk (Fahlman et al., 2006; Hooker et al., 2009; Kvadsheim et al., 2012). The results presented by Kvadsheim et al. (2012) implied a relationship between dive depth and the risk of gas bubble emboli, as the end-dive P_N_2__ and instantaneous risk increased during deeper dives. However, deep divers perform dives that are significantly deeper than 200 m, where the pulmonary shunt prevents any significant gas exchange. We propose that it is not the maximum depth that increases the risk of gas bubble emboli in deep divers, but a complex relationship between dive duration and the time spent at intermediate depths (approximated between 30 and 200 m), where N_2_ is being absorbed, vs. time spent at shallow depths (0–30 m) closer to the surface where N_2_ can be removed. Consequently, dive depth and duration by themselves may not be good indicators of the risk of emboli formation.

This interpretation is supported by the result of Kvadsheim et al. (2012) which showed that pilot whales, which make relatively short duration deep dives beyond the expected depth of alveolar collapse (Aguilar Soto et al., 2008; Sivle et al., 2012), have end-dive N_2_ levels, and thereby DCS risk, which is comparable to killer whales that make only short and shallow dives (Sivle et al., 2012). Deep divers such as sperm whales (Teloni et al., 2008; Sivle et al., 2012) and beaked whales (Hooker and Baird, 1999; Baird et al., 2006, 2008) conduct longer duration dives. During such deep dives, less time is spent at shallow depths where N_2_ would be removed, and more time is spent in the intermediate depth zone where N_2_ is taken up as compared with shallow divers, such as killer whales (Figure 1). The extended dive duration, even though it is deeper than the expected depth of alveolar collapse, allows redistribution of N_2_ into slow tissues, resulting in gradual N_2_ accumulation in these tissues (Fahlman et al., 2007; Hooker et al., 2009).

For marine mammals that dive in bouts with repeated dives separated by much shorter surface intervals, such as elephant seals and sperm whales, the amount of inert gas that is taken up may accumulate across dives to levels that may cause bubble growth (Fahlman et al., 2006, 2007, 2009; Hooker et al., 2009). While N_2_ from fast tissues, such as the heart, is rapidly removed as the whale approaches the surface, this continuous buildup of N_2_ in adipose tissue may eventually become a liability and increase the risk of bubble formation and growth. It has been suggested that the high N_2_ capacitance of adipose tissues would act as a scrubber and reduce bubble formation during deep, short duration dives (Behnke et al., 1935; Fahlman et al., 2007). However, if the adipose P_N_2__ accumulates over repeated dives, it could become a liability after an extended dive bout. Elevated levels of adipose P_*N*_2__ may be one reason why deep foraging dive bouts sometimes are terminated at times when food is most available and separated in time by series of short and shallow decompression dives (Hooker et al., 2009; Fahlman et al., 2007). It may be that the effect of alveolar collapse is a lot more complicated than formerly thought, and that the redistribution of inert gas between tissues with different time constants contribute to the risk in some species.

In summary we have identified three main risk factors that potentially explain why deep divers have an elevated risk of developing gas bubble embolism compared to shallow divers:

1. The longer duration dives combined with exercise during dives will increase the tissue CO_2_-levels, and this could initiate bubble growth in a supersaturated body.
2. Deep divers may spend more time in the intermediate (compression) zone where N_2_ is absorbed during descent and ascent.
3. The longer duration of deep dives allows for transfer of N_2_ from fast to slow tissues (e.g., fat), which leads to N_2_ accumulation.

Can anthropogenic disturbances further increase risk of bubble embolism in deep divers? Bubbles have been seen in live stranded dolphins (Dennison et al., 2012) and marine mammals may have physiological and behavioral traits to manage a certain bubble load (Hooker et al., 2012). However, if an animal is disturbed and deviates substantially from its normal diving behavior or physiological balance, the supersaturation may reach a critical level and result in symptomatic bubbles (Hooker et al., 2012). Cuvier's beaked whales escape from naval sonar with vigorous swimming and reduced surface durations (Deruiter et al., 2013), and the behavior of this species is consistent with the observation that it is particularly prone to **strandings** following military exercises (D'amico et al., 2009). Similarly, sperm whales might respond to sonar by performing deep dives that are shallower than usual and increasing the time spent in the intermediate depth range where N_2_ is absorbed (Kvadsheim et al., 2012). Thus, both sperm whales (Sivle et al., 2012) and beaked whales (Tyack et al., 2011; Deruiter et al., 2013) respond to naval sonar by altering dive behavior in a way that we have identified as behavioral factors increasing risk of gas bubble embolism. Thus, anthropogenic noise may alter both dive behavior and underwater activity, which in turn may affect the risk of bubble formation. However, a better understanding of the physiological responses are also required to determine how to mitigate the risk of gas bubble emboli in deep diving whales.

KEY CONCEPT 5. Stranding Beaching of cetaceans, most commonly odontocetes (toothed whales). A mass-stranding involves 2 or more whales (excluding mother calf pairs). Strandings may be caused by disease, rough weather, old age, navigation errors. In some cases human interaction may cause cetaceans to strand.

Nothing is known about additional physiological responses during these events, but the apparently complex interaction between dive behavior (e.g., changes in time spent at depth) and physiological factors such as variation in cardiac output and/or blood flow distribution may imply that deep divers are highly susceptible to anthropogenic disturbances. If CO_2_ is implicated in the genesis of bubbles, by diving for longer or being more active during the dive, elevated levels of CO_2_ may initiate bubble formation. Recent research has focused on revealing the behavioral responses to anthropogenic noise, and this research has allowed us to simulate potential consequences of behavioral responses in changing risk of bubble formation in cetaceans (Kvadsheim et al., 2012). There is an equal need for studies on physiological responses of deep divers whose behavior is disturbed by anthropogenic noise or other threats. Particularly important is an improved understanding of how pressure affects gas exchange and patterns of cardiac output and blood flow distribution during submersion. Effective mitigation of negative impact on marine life requires a better understanding of this link.

### Conflict of interest statement

The authors declare that the research was conducted in the absence of any commercial or financial relationships that could be construed as a potential conflict of interest.
